# UK Medical Cannabis Registry: a case series analysing clinical outcomes of medicinal cannabis therapy for fibromyalgia

**DOI:** 10.1007/s10067-025-07846-6

**Published:** 2025-12-04

**Authors:** Madhur Varadpande, Simon Erridge, Arushika Aggarwal, Evonne Clarke, Katy McLachlan, Ross Coomber, Shelley Barnes, Alia Darweish Medniuk, Rahul Guru, Wendy Holden, Mohammed Sajad, Robert Searle, Azfer Usmani, Sanjay Varma, James J. Rucker, Michael Platt, Mikael H. Sodergren

**Affiliations:** 1 Medical Cannabis Research Group, Department of Surgery & Cancer, Imperial College London, Academic Surgical Unit, St Mary’s Hospital, 10Th Floor QEQM, St Mary’s Hospital, South Wharf Road, London, W2 1NY UK; 2 Curaleaf Clinic, London, UK; 3 St. George’s Hospital NHS Trust, London, UK; 4 North Bristol NHS Trust, Bristol, UK; 5 Vale University Health Board, Cardiff, UK; 6 Department of Psychological Medicine, Kings College London, London, UK; 7 South London & Maudsley NHS Foundation Trust, London, UK

**Keywords:** Cannabidiol, Cannabinoid, Cannabis, Fibromyalgia, Tetrahydrocannabinol

## Abstract

**Introduction:**

Fibromyalgia is a common condition characterised by widespread chronic pain, associated with comorbid mental health disorders and reduced quality of life. Preclinical data suggest cannabis-based medicinal products (CBMPs) may have potential benefits in fibromyalgia, but there is a paucity of high-quality clinical evidence. This study aims to assess the change in patient-reported outcome measures (PROMs) and incidence of adverse events (AEs) in patients treated with CBMPs for fibromyalgia.

**Methods:**

This case series analysed data from the UK Medical Cannabis Registry (UKMCR). The primary outcome was change in PROMs [Fibromyalgia Symptom Severity, Fibromyalgia Widespread Pain Index, EQ-5D-5L, Generalised Anxiety Disorder-7, and Single-Item Sleep Quality Scale] from baseline to follow-up at 1, 3, 6, 12, and 18 months. Statistical significance was defined as *p* < 0.050.

**Results:**

Four hundred ninety-seven patients were included. The mean age was 44.66 ± 12.02 years, 341 patients (68.61%) were female, and the majority of patients were unemployed (*n* = 268, 53.92%). There was an improvement in all PROMs (*p* < 0.010) from baseline to all follow-up periods. Higher CBD doses (> 25.00 mg/day) and previous cannabis use were associated with increased odds of improvement on fibromyalgia-specific scales (*p* < 0.050). 227 patients (45.67%) reported 2100 AEs (422.54%). Most AEs were mild-to-moderate (*n* = 1792, 85.33%). The most common AE was fatigue (*n* = 153, 30.78%).

**Conclusions:**

There was an association between treatment with CBMPs and improvements in pain, anxiety, sleep, and general quality of life. The high incidence of AEs in relation to other patient cohorts from the UKMCR may relate to the central sensitisation mechanism of fibromyalgia.

**Supplementary information:**

The online version contains supplementary material available at 10.1007/s10067-025-07846-6.

**Table:** 

**Key Points** *• This study found that CBMPs were associated with short to medium-term improvements in pain, anxiety, sleep, and general quality-of-life in patients with fibromyalgia.* *• More randomised controlled trials are warranted to consolidate the literature, but this large analysis provides real-world data to inform their rollout.*	

## Introduction

Fibromyalgia is defined as generalised pain lasting for at least three months, and it is typically accompanied by tender points or somatic symptoms, such as fatigue, unrefreshed sleep, and cognitive problems [1]. Fibromyalgia is estimated to affect 5.4% of adults and is more than twice as prevalent in females [2]. The pain is hypothesised to manifest through nociplastic mechanisms, where sensitisation in the central nervous system results in a dysfunctional, heightened pain response [3]. Fibromyalgia carries a significant socioeconomic burden, with 1 in 2 patients affected in their work [4]. Quality of life can be extremely low, worsening existing mental health states [4]. Over half of patients have comorbid insomnia (68%) and depressive symptoms (58%) [4].

There is no cure for fibromyalgia, but treatment can alleviate symptoms and improve function. First-line pharmacological options include pregabalin, an anti-convulsant, and anti-depressants including duloxetine, amitriptyline, and milnacipran. Whilst pregabalin, duloxetine, and milnacipran are effective, the effect sizes are small [5]. This, in combination with poor tolerability of adverse events (AEs), leads to high rates of medication discontinuation [5, 6], with one study suggesting non-adherence rates may be as high as 72.5% [7]. The need for additional pharmacotherapeutic options draws interest to novel medications, such as cannabis-based medicinal products (CBMPs).

The most prevalent active pharmaceutical ingredients in CBMPs are delta-9-tetrahydrocannabinol (THC) and cannabidiol (CBD). THC is a partial agonist at the cannabinoid-1 receptor (CB_1_R) and cannabinoid-2 receptor (CB_2_R). CBD reduces the breakdown of the endogenous cannabinoid anandamide, which acts as an agonist at the CB_1_R and CB_2_R [8]. Preclinical data have shown activation of the CB_1_R and CB_2_R has analgesic effects [9]. Additionally, there are potential sleep-promoting and anxiolytic effects through the CB_1_R [10].

It is estimated that 1 in 2 patients with fibromyalgia consume cannabis, and a cross-sectional study found that in this group, 82% experience an improvement in pain [11]. However, there is a lack of consensus on the effectiveness of CBMPs in treating fibromyalgia due to a paucity of high-quality randomised controlled trials (RCTs). A 2021 meta-analysis of randomised controlled trials (RCTs) estimated that non-inhaled CBMPs are associated with clinically significant pain relief in a small proportion of chronic pain patients, compared to placebo [12]. This led to a weak recommendation to trial non-inhaled CBMPs for chronic pain if standard care is insufficient [13]. However, the meta-analysis upon which this recommendation is based only included one RCT of fibromyalgia [12]. Canadian guidelines for the management of fibromyalgia, meanwhile, provide a weak recommendation that CBMPs may be considered in fibromyalgia, especially when it is accompanied by impaired sleep [14]. Two recent systematic reviews in fibromyalgia patients found supporting evidence for CBMPs in short-term pain reduction [15, 16]. There are four RCTs in the field, with two supporting the use of CBMPs in addressing pain caused by fibromyalgia [17, 18]. A further two RCTs comparing nabilone and CBMP oils to amitriptyline and placebo, respectively, show mixed findings [19, 20]. However, interpretation of the effects of CBMPs is affected by small sample size, limited follow-up, and significant heterogeneity. Notably, there has only been one trial investigating CBMPs via an inhaled route. Yet, up to 60% of chronic pain patients prescribed CBMPs administer them through inhalation [21], suggesting a disconnect between clinical studies and real-world patterns, highlighting a pressing need for further research.

Previous prospective observational studies of the UK Medical Cannabis Registry (UKMCR) have found CBMPs to be associated with improvements in fibromyalgia-specific and general-health measures across 12 months, with mostly mild–moderate adverse events (AEs) [22, 23]. This study seeks to update the literature with a larger sample size and longer-term follow-up from baseline. This study primarily aims to assess the change in patient-reported outcome measures (PROMs) of patients enrolled in the UKMCR who are prescribed CBMPs for fibromyalgia. The secondary aim is to assess the prevalence of AEs in this group of patients.

## Methods

### Study design

This case series analysed clinical data from the UKMCR for patients prescribed CBMPs for fibromyalgia. Patients completed PROMs at baseline and follow-up intervals of 1, 3, 6, 12, and 18 months, and reported AEs.

A favorable ethical opinion was confirmed for the UKMCR from the Health Research Authority (Central Bristol Research Ethics Committee reference: 22/SW/0145). The reporting of this study adheres to the Strengthening the Reporting of Observational Studies in Epidemiology recommendations [24].

### Settings and participants

Established in December 2019, the UKMCR holds prospectively collected, pseudonymised clinical data on CBMP outcomes in patients from the UK and Crown Dependencies. The UKMCR is the UK’s largest platform of its kind and is privately managed and owned by Curaleaf Clinic. All patients provide written, informed consent prior to enrollment with the registry and the commencement of data collection.

Patients were included in this study upon fulfilment of the following criteria: (1) confirmation of a primary diagnosis of fibromyalgia by a consultant physician; (2) enrolment in the UKMCR ≥ 18 months prior to data extraction; and (3) completion of a minimum of one baseline PROM questionnaire. Patients were excluded if their primary diagnosis was not fibromyalgia.

### Cannabis-based medical products

All unlicensed CBMP prescriptions in the UK comply with regulations from the Medicines and Healthcare Products Regulatory Agency. Patients with fibromyalgia are only eligible to be prescribed CBMPs if they have failed to gain sufficient improvement in symptoms from licensed therapies [25]. They are then reformulated into medium-chain triglyceride oils or formulated into capsules, pastilles, and lozenges, administered sublingually or orally. Dried flower is inhaled through a vaporisation device. Patients were strongly counselled to discourage the use of any non-prescribed cannabis at baseline.

### Data collection

Baseline demographic data was collected, including age, gender, height, weight, body mass index (BMI), occupation, comorbidities, and area of residence. Charlson-Comorbidity Index (CCI) values were calculated individually for patients. The CCI is a validated predictor of a patient’s long-term prognosis and survival [26]. Tobacco, alcohol, and cannabis use histories were also collected. Tobacco and cannabis status options included: ex-user, current user, or never used. Alcohol consumption was measured in weekly units. Lifetime tobacco use was quantified in pack-years [27], whilst cannabis use was measured in ‘gram-years’ (daily grams × years consumed) [28]. Clinicians and pharmacists documented CBMP details, including manufacturer, formulation, THC/CBD concentrations, and dosages, cannabis strain, and administration method.

All participants completed baseline PROM questionnaires and were sent follow-up PROM questionnaires at 1, 3, 6, 12, and 18 months. For any missing PROM data, the baseline observations carried forward method was used, which assumes no improvement from baseline measures, hence providing a conservative measure of true effects. Patients self-recorded any AEs, either just before completing PROMs via a bespoke electronic portal or contemporaneously in an online form. Clinicians also recorded any AEs reported during follow-up consultations. AEs were classified and graded in accordance with the Common Terminology Criteria for AEs Version 4.0 [29].

### Outcome measures

The primary outcomes were changes in PROMs from baseline to 1, 3, 6, 12, and 18 months in all participants. The secondary outcome was the incidence of AEs in all participants.

Fibromyalgia-specific scales include the 12-point Symptom Severity (SS) scale, which evaluates three core symptoms (fatigue, unrefreshed sleep, cognitive symptoms) rated 0–3 each, wherein ‘0’ indicates no problem and ‘3’ indicates severe. In addition, 1 point is given for the presence of each of the following three symptoms: abdominal cramps, depression, and headache. The 19-point Widespread Pain Index (WPI) assesses pain/tenderness across 19 body areas, scoring 1 point per affected area. A fibromyalgia diagnosis requires a SS ≥ 5 or WPI ≥ 7, with symptoms persisting ≥ 3 months and no alternative explanation for pain [30].

The European Quality-of-Life 5 Dimension–5 Levels scale (EQ-5D-5L) evaluates five domains of health-related quality-of-life: anxiety/depression, mobility, pain/discomfort, self-care, and usual activities. Patients rate each domain from ‘1’, indicating no problems, to ‘5’, indicating extreme problems. From the scores in each domain, an index score is calculated. A value of ‘1’ signifies “full health” and a value of ‘ < 0’ signifies “worse than death”.

The Generalised Anxiety Disorder scale (GAD-7) assesses how frequently seven different anxiety symptoms have occurred in the past two weeks [31]. The severity of anxiety is categorised with scores as minimal (0–4), mild (5–9), moderate (10–14), and severe (15–21).

Patient Global Impression of Change (PGIC) is a 7-point numerical rating scale, allowing patients to report their improvement [32]. A score of ‘1’ signifies “no change or condition has worsened” and ‘7’ signifies “considerable improvement”.

The Single-Item Sleep Quality Scale (SQS) is a single-item numerical rating scale, allowing patients to rate their overall sleep quality over the past week [33]. It is scored from ‘0’, representing “terrible”, to ‘10’, representing “excellent”.

### Statistical analysis

Baseline patient demographic details were reported using descriptive statistics. Parametric and non-parametric data were presented as mean ± standard deviation (SD) and median [interquartile range (IQR)], respectively. To assess the primary outcomes of changes in PROMs, a repeated measures analysis of variance (ANOVA) with Greenhouse–Geisser correction was performed. If values on the repeated measures ANOVA were statistically significant, post-hoc multiple pairwise comparisons were conducted, with Bonferroni correction to reduce type 1 error. To assess the secondary outcome of incidence of AEs, descriptive statistics were used.

To evaluate treatment and patient-specific factors linked to a clinically significant pain reduction, univariate and multivariate logistic regression analyses were conducted. In univariate analyses, individual logistic regression models were created for each independent variable of interest, against a positive improvement in a fibromyalgia-related outcome measure. The models generated odds ratios with corresponding 95% confidence intervals to quantify the influence of each factor in achieving the MCID. As variables were intrinsically linked, all variables were carried forward into a multivariate logistic regression analysis.

All statistical analyses were conducted using the Statistical Packages for the Social Sciences version 28.0 (IBM SPSS Statistics for Macintosh, Version 28.0 Armonk, NY: IBM Corp). Statistical significance was defined as *p-*value < 0.050.

## Results

### Patient data

**Fig. 1:**
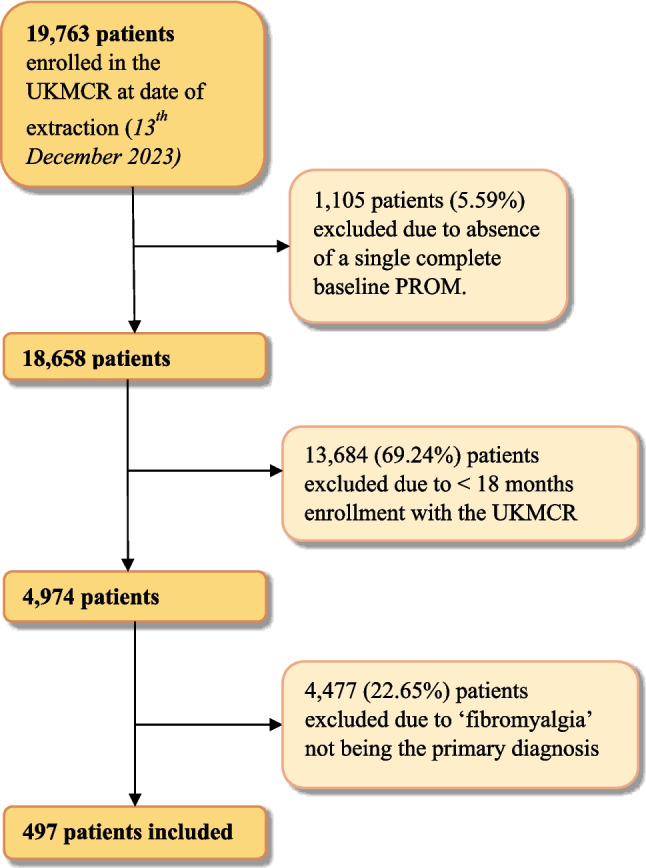
A flowchart summarising patient inclusion and exclusion criteria for this study. Abbreviations: UKMCR, UK Medical Cannabis Registry; PROM, patient-reported outcome measure

At data extraction, on the 13th of December 2023, there were 19,763 patients enrolled in the UKMCR. Following the application of inclusion and exclusion criteria, 497 patients were included in the final analysis (Fig. 1*)*. Reasons for patient exclusion included not having completed any baseline PROM questionnaires (*n* = 1,105, 5.59%), enrolled in the UKMCR less than 18 months prior to data extraction (*n* = 13,684, 69.24%), and if the primary diagnosis was not ‘fibromyalgia’ (*n* = 4477, 22.65%).

Patient baseline demographics are presented in Table 1*.* Out of all 497 patients, 341 (68.61%) were female, 154 (30.99%) were male, and 2 (0.40%) identified as another gender. The mean age was 44.66 ± 12.02 years, and the mean BMI was 29.47 ± 8.64 kg/m^2^. The median CCI was 1.00 [0.00–6.00]. All patient comorbidities are listed in Appendix A***.*** The most prevalent co-morbidity was depression or anxiety (*n* = 263, 52.92%). The largest occupation category was unemployed (*n* = 268, 53.92%).

**Table 1:** Demographic details of all participants is shown

Baseline demographics	*n* (%)/mean ± SD/median [IQR]
Gender	
Male	154 (30.99)
Female	341 (68.61)
Other	2 (0.40)
Age (years)	44.66 ± 12.02
Height (cm)	167.93 ± 9.79
Weight (kg)	83.35 ± 25.67
BMI (kg/m^2^)	29.47 ± 8.64
Charlson Comorbidity Index	1.00 [0.00–6.00]
Occupation status	
Employed	212 (42.66)
Retired	1 (0.20)
Unemployed	268 (53.92)
Unknown	16 (3.22)
Weekly alcohol consumption (units)	0.00 [0.00–2.00]
Tobacco status	
Current smoker	147 (29.58)
Ex-smoker	209 (42.05)
Never smoked	141 (28.37)
Lifetime tobacco consumption (pack years)	10.00 [5.00–20.00]
Cannabis status	
Current user	264 (53.12)
Ex-user	77 (15.49)
Never used	156 (31.39)
Cannabis daily consumption (grams)	1.00 [1.00–2.00]
Frequency of cannabis consumption	
Daily	230 (46.28)
Every other day	21 (4.23)
1–2 times per week	7 (1.41)
> 1 times per month	2 (0.40)
< 1 times per month	4 (0.80)
Lifetime cannabis consumption (gram years)	5.00 [1.55–18.00]

*BMI*, body mass index; *CCI*, Charlson Comorbidity Index; *n*, number of participants; *%*, percentage; *SD*, standard deviation; *IQR*, interquartile range; *cm*, centimetres; *kg*, kilograms; *m*^*2*^, metres squared of patients at baseline assessment

Baseline alcohol, tobacco, and cannabis consumption is also presented in Table 1. The median weekly alcohol consumption was 0.00 [0.00–2.00] units. 209 patients (42.05%) were ex-smokers, 147 (29.58%) currently smoke, and 141 (28.37%) have never smoked. The median lifetime tobacco consumption was 10.00 [5.00–20.00] pack years. At baseline, 264 patients (53.12%) reported being current users of cannabis, 77 (15.49%) were ex-users, and 156 (31.39%) reported they had never used cannabis. Of those who had used cannabis, the median lifetime cannabis consumption was 10.00 [5.00–20.00] gram years. Patients originated from all areas of the UK and from the Channel Islands, and full details for this are presented in Appendix B.

### Cannabis-based medicinal product details

Table 2 presents data regarding CBMPs prescribed at baseline and follow-up intervals of 1 month, 3 months, 6 months, 12 months, and 18 months. At baseline, most patients (*n* = 280, 56.34%) administered CBMPs as oils alone, but this value decreased over time, with most patients at follow-up at 18 months administering CBMPs as oils and dry flower (*n* = 249, 50.10%). The median CBD dosage increased slightly from 20.00 [20.00–20.00] mg/day at baseline to 25.00 [20.00–52.50] mg/day at 18 months. The median THC dosage greatly increased from a baseline measure of 2.00 [1.00–21.00] mg/day to 112.50 [15.80–216.80] mg/day at the 18-month follow-up. Adven EMC1 50/< 4 mg/ml CBD/THC (Curaleaf International, UK) and Adven EMT 20 mg/ml THC (Curaleaf International, UK) were the most frequently prescribed CBD- and THC-dominant oils. The most commonly prescribed dried flower was Adven EMT2 16%/< 1% THC/CBD (Curaleaf International, UK).

**Table 2:** Details of cannabis-based medicinal products prescribed to patients at baseline and follow-up at 1 month (*n* = 497), 3 months (*n* = 497), 6 months (*n* = 497), 12 months (*n* = 497), and 18 months (*n* = 497)

*n* (%)/median [IQR]						
	Baseline	Follow-up at 1 month	Follow-up at 3 months	Follow-up at 6 months	Follow-up at 12 months	Follow-up at 18 months
Administration						
None	0 (0.00)	2 (0.40)	4 (0.80)	8 (1.61)	8 (1.61)	4 (0.80)
Oils	280 (56.34)	242 (48.69)	208 (41.85)	181 (36.42)	149 (29.98)	147 (29.58)
Dry flower	43 (8.65)	42 (8.45)	49 (9.86)	68 (13.68)	88 (17.71)	97 (19.52)
Oils and dry flower	174 (35.01)	211 (42.45)	236 (47.48)	240 (48.29)	252 (50.70)	249 (50.10)
Dosage (milligrams/day)						
CBD	20.00 [20.00–20.00]	20.00 [20.00–20.00]	20.00 [20.00–30.00]	20.00 [20.00–38.13]	20.00 [10.00–50.00]	25.00 [20.00–52.50]
THC	2.00 [1.00–21.00]	40.00 [5.00–110.00]	100.00 [10.00–115.00]	103.94 [10.00–194.75]	110.00 [13.53–205.36]	112.50 [15.80–216.80]

*n*, number of participants; *IQR*, interquartile range; *CBD*, cannabidiol; *THC*, delta-9-tetrahydrocannabinol

### Patient-reported outcome measures

Table 3 presents a comparison of the mean PROM score across all follow-up periods with a repeated-measures one-way ANOVA. All PROMs demonstrated a change (*p* < 0.001). As a result, post-hoc pairwise comparisons were conducted for all PROMs, with Bonferroni correction to adjust for multiple testing.

**Table 3:** Patient-reported outcome measures (PROMs) at baseline, 1 month, 3 months, 6 months, 12 months, and 18 months. Patient global impression of change (PGIC) has no baseline measure due to the nature of the scale

	PROM	Baseline	1 month	3 months	6 months	12 months	18 months	*p*-value
Fibromyalgia-specific scales	Fibromyalgia Symptom Severity	9.08 ± 2.19	7.92 ± 2.42	7.96 ± 2.58	8.05 ± 2.59	8.24 ± 2.58	8.36 ± 2.54	< 0.001***
	Fibromyalgia Widespread Pain Index	13.96 ± 4.02	12.21 ± 4.56	12.26 ± 4.55	12.61 ± 4.78	12.81 ± 4.44	12.95 ± 4.43	< 0.001***
General Health Assessment Scales	EQ-5D-5L Anxiety and Depression	2.82 ± 1.19	2.51 ± 1.10	2.52 ± 1.15	2.54 ± 1.15	2.59 ± 1.13	2.62 ± 1.19	< 0.001***
	EQ-5D-5L Mobility	3.01 ± 0.97	2.79 ± 0.94	2.82 ± 0.99	2.82 ± 1.00	2.85 ± 0.99	2.90 ± 0.95	< 0.001***
	EQ-5D-5L Pain and Discomfort	3.88 ± 0.83	3.34 ± 0.92	3.34 ± 0.94	3.40 ± 0.97	3.47 ± 0.93	3.52 ± 0.95	< 0.001***
	EQ-5D-5L Self-Care	2.50 ± 1.01	2.33 ± 1.03	2.36 ± 1.01	2.36 ± 1.01	2.35 ± 0.98	2.38 ± 0.98	< 0.001***
	EQ-5D-5L Usual Activities	3.31 ± 0.97	2.89 ± 1.02	2.91 ± 1.01	2.94 ± 1.10	3.03 ± 1.03	3.04 ± 1.02	< 0.001***
	EQ-5D-5L Index Values	0.25 ± 0.30	0.40 ± 0.29	0.39 ± 0.30	0.37 ± 0.31	0.36 ± 0.30	0.34 ± 0.31	< 0.001***
	GAD-7	9.68 ± 6.38	7.69 ± 5.75	8.00 ± 6.04	8.18 ± 6.16	8.21 ± 6.13	8.41 ± 6.22	< 0.001***
	PGIC		4.85 ± 1.51	5.03 ± 1.47	5.13 ± 1.44	5.15 ± 1.45	5.18 ± 1.47	< 0.001***
	SQS	3.42 ± 2.29	4.83 ± 2.57	4.68 ± 2.63	4.52 ± 2.58	4.40 ± 2.55	4.32 ± 2.57	< 0.001***

Significance values are shown as: ‘***’: *p* < 0.001; ‘**’: *p* < 0.010; ‘*’: *p* < 0.050

*PROM*, patient-reported outcome measure; *EQ-5D-5L*, European Quality-of-Life 5 Dimension–5 levels; *GAD-7*, Generalised Anxiety Disorder scale; *PGIC*, patient global impression of change; *SQS*, single-item sleep quality scale

The results of post-hoc pairwise comparisons are presented in Table 4. All scales, including Fibromyalgia SS, Fibromyalgia WPI, EQ-5D-5L, GAD-7, and SQS, showed an improvement (*p* < 0.010) from baseline to follow-up periods of 1, 3, 6, 12, and 18 months. The Fibromyalgia SS mean score saw the greatest improvement from baseline at a follow-up of 1 month by 1.16 ± 0.09 (*p* < 0.001), with the least improvement from baseline at 18 months by 0.72 ± 0.09 (*p* < 0.001). A similar trend was seen in all other PROMs.

**Table 4:** Pairwise comparison of patient-reported outcome measures (PROMs) taken at baseline and change in the PROM value at follow-up at 1 month, 3 months, and 6 months

	Patient-reported outcome measure	Mean baseline score ± SD	*n*	Follow-Up (months)	*n*	Mean difference from baseline ± SD	*p*-value
Fibromyalgia-specific scales	Fibromyalgia Symptom Severity	9.08 ± 2.19	446	1	446	1.16 ± 0.09	< 0.001***
				3	446	1.12 ± 0.10	< 0.001***
				6	446	1.02 ± 0.10	< 0.001***
				12	446	0.84 ± 0.09	< 0.001***
				18	446	0.72 ± 0.09	< 0.001***
	Fibromyalgia Widespread Pain Index	13.96 ± 4.02	446	1	446	1.75 ± 0.17	< 0.001***
				3	446	1.71 ± 0.18	< 0.001***
				6	446	1.36 ± 0.18	< 0.001***
				12	446	1.15 ± 0.17	< 0.001***
				18	446	1.02 ± 0.16	< 0.001***
General health assessment scales	EQ-5D-5L Anxiety and Depression	2.82 ± 1.19	497	1	497	0.31 ± 0.04	< 0.001***
				3	497	0.31 ± 0.04	< 0.001***
				6	497	0.28 ± 0.04	< 0.001***
				12	497	0.24 ± 0.04	< 0.001***
				18	497	0.20 ± 0.04	< 0.001***
	EQ-5D-5L Mobility	3.01 ± 0.97	497	1	497	0.22 ± 0.04	< 0.001***
				3	497	0.19 ± 0.04	< 0.001***
				6	497	0.20 ± 0.04	< 0.001***
				12	497	0.16 ± 0.04	< 0.001***
				18	497	0.11 ± 0.03	0.002**
	EQ-5D-5L Pain and Discomfort	3.88 ± 0.83	497	1	497	0.54 ± 0.04	< 0.001***
				3	497	0.53 ± 0.04	< 0.001***
				6	497	0.48 ± 0.04	< 0.001***
				12	497	0.41 ± 0.04	< 0.001***
				18	497	0.36 ± 0.04	< 0.001***
	EQ-5D-5L Self-Care	2.50 ± 1.01	497	1	497	0.18 ± 0.03	< 0.001***
				3	497	0.15 ± 0.03	< 0.001***
				6	497	0.14 ± 0.03	< 0.001***
				12	497	0.15 ± 0.03	< 0.001***
				18	497	0.13 ± 0.03	< 0.001***
	EQ-5D-5L Usual Activities	3.31 ± 0.97	497	1	497	0.42 ± 0.04	< 0.001***
				3	497	0.40 ± 0.04	< 0.001***
				6	497	0.37 ± 0.04	< 0.001***
				12	497	0.29 ± 0.04	< 0.001***
				18	497	0.27 ± 0.03	< 0.001***
	EQ-5D-5L Index Values	0.25 ± 0.30	497	1	497	0.15 ± 0.01	< 0.001***
				3	497	0.13 ± 0.01	< 0.001***
				6	497	0.12 ± 0.01	< 0.001***
				12	497	0.11 ± 0.01	< 0.001***
				18	497	0.09 ± 0.01	< 0.001***
	Generalised Anxiety Disorder-7 scale	9.68 ± 6.38	497	1	497	1.99 ± 0.23	< 0.001***
				3	497	1.68 ± 0.23	< 0.001***
				6	497	1.50 ± 0.23	< 0.001***
				12	497	1.47 ± 0.20	< 0.001***
				18	497	1.27 ± 0.18	< 0.001***
	Single-Item Sleep Quality Scale	3.42 ± 2.29	496	1	496	1.41 ± 0.12	< 0.001***
				3	496	1.26 ± 0.11	< 0.001***
				6	496	1.10 ± 0.11	< 0.001***
				12	496	0.98 ± 0.10	< 0.001***
				18	496	0.90 ± 0.10	< 0.001***

Significance values are shown as: ‘***’: *p* < 0.001; ‘**’: *p* < 0.01; ‘*’: *p* < 0.05

*SD*, standard deviation; *n*, number of participants; *EQ-5D-5L*, European Quality-of-Life 5 Dimension–5 levels

Univariate logistic regression analyses on factors associated with an improvement in fibromyalgia-specific scales are contained in Appendices D, F, and H. Multivariate logistic regression confirmed that a high CBD dose was associated with increased odds of an improvement in the Fibromyalgia SS score (OR = 1.64; 95% CI: 1.04–2.61; *p* = 0.035), the fibromyalgia WPI (OR = 1.90; 95% CI: 1.19–3.03; *p* = 0.007) and fibromyalgia overall score (OR = 2.42; 95% CI: 1.51–3.86; *p* < 0.001) (Appendices E, G, I). Being a current user of cannabis was associated with an increased likelihood for improvement in the Fibromyalgia SS score (OR = 2.32 95% CI: 1.22–4.43; *p* = 0.011), the fibromyalgia WPI (OR = 2.32; 95% CI: 1.21–4.45; *p* = 0.011) and overall score (OR = 1.97; 95% CI: 1.04–3.73; *p* = 0.038). Furthermore, the patient age group of 41–50 years was associated with increased odds of improvements in the fibromyalgia WPI scale (OR = 3.39; 95% CI: 1.38–8.31; *p* = 0.008) and fibromyalgia scale overall (OR = 2.95; 95% CI: 1.26–6.93; *p* = 0.013). Higher THC doses were no longer associated with increased odds of improvement on any score (*p* > 0.050).

### Adverse events

As shown in Appendix D, 227 patients (45.67%) reported 2100 AEs (422.54%). Most AEs were mild-to-moderate (*n* = 1792, 85.33,%); 306 (14.57%) were severe, and 2 (0.10%) were life-threatening. Life-threatening AEs included anxiety (*n* = 1, 0.05%) and lung infection (*n* = 1, 0.05%). The most common AE was fatigue (*n* = 153, 30.78%), followed by dry mouth (*n* = 137, 27.57%) and concentration impairment (*n* = 130, 26.16%).

Univariate logistic regression analyses on factors associated with increased odds of patients reporting an AE are contained in Appendix J*.* On multivariate logistic regression, patients were less likely to report an AE if they were 41–50 years old (OR = 0.40; 95% CI: 0.18–0.88; *p* = 0.023), a current user of cannabis ((OR = 0.46; 95% CI: 0.26–0.83; *p* = 0.010), administering CBMPs as both oils and dry flower (OR = 0.44.; 95% CI: 0.22–0.90; *p* = 0.025) and having poor sleep at baseline (OR = 0.47; 95% CI: 0.29–0.75; *p* = 0.002) (Appendix K). The age group 31–40 years old was no longer associated with a lower likelihood to report and AE, but multivariate analysis found being aged over 71 to be associated with lower odds of reporting an AE (OR = 0.09; 95% CI: 0.01–0.84; *p* = 0.034). Multivariate analysis also found a high CBD dose to increase the odds of reporting an AE (OR = 2.28; 95% CI: 1.42–3.66; *p* = < 0.001), as well as moderate baseline anxiety (OR = 2.24; 95% CI: 1.16–4.32; *p* = 0.017) and severe baseline anxiety (OR = 1.94; 95% CI: 1.01–3.70; *p* = 0.045).

## Discussion

This prospective case series assessed the change in PROMs and AEs for patients prescribed CBMPs for fibromyalgia. CBMPs were associated with improvements in all PROMs, fibromyalgia-specific and general-health related, from baseline to all follow-up measures at 1, 3, 6, 12, and 18 months. In comparison to previous studies from the UKMCR of other conditions, a larger proportion of patients (45.67%) reported AEs [21, 34]. But similarly, most AEs were classified as mild-to-moderate severity (85.33%). Patients were more likely to report an improvement in fibromyalgia-specific scales if they were prescribed a higher CBD dose (> 25.00 mg/day), were current users of cannabis, or were between 41 and 50 years old.

### Patient-reported outcome measures

The improvement in fibromyalgia-specific PROMs is supported by an RCT by Skrabek et al*.* of 40 patients, which found that nabilone, a synthetic cannabinoid, improved symptoms on the fibromyalgia impact questionnaire (FIQ) at 4 weeks, but not at 2 weeks with a lower dose, compared to placebo [17]. This is in accordance with findings from the present study that higher CBD doses were associated with greater odds of improved outcomes. However, the RCT was limited by its short 4-week duration. A longer 8-week RCT by Chaves et al*.* demonstrated significant FIQ improvement in 17 patients taking CBMPs compared to placebo and baseline [18]. Patients in this RCT had a higher baseline FIQ than the study by Skrabek et al*.*, indicating worsening baseline symptoms, but experienced a greater reduction in FIQ score and saw the greatest improvement in the ‘feel good’ section. Interestingly, a THC-rich oil was used in this trial, and THC is thought to impact mood through agonist activity at CB_1_R [35].

Conversely, an RCT by van de Donk et al*.* found CBMPs did not reduce spontaneous or electrical pain compared to placebo [20]. However, two formulations, Bedrocan (22.4 mg THC, < 1 mg CBD) and Bediol (13.4 mg THC, 17.8 mg CBD), increased the pressure pain threshold and caused euphoria as measured by the Bowdle questionnaire. More patients using Bediol achieved a 30% reduction in spontaneous pain compared to placebo [20]. The reduction in pain severity was positively correlated with the experience of euphoria. This suggests the psychoactive effects of CBMPs may help via altering the cognitive and emotional interpretation of pain. Whilst the RCT by van de Donk et al*.* is valuable in investigating inhaled CBMPs, its findings are limited to acute settings as patients received only a single dose [20].

This study found improvements in fibromyalgia-specific PROMs at a long-term follow-up of 18 months, although these gradually declined over time, with peak improvement from baseline seen at 1 month and lowest at 18 months. A previous UKMCR fibromyalgia analysis showed similar trends [23], possibly due to cannabis-induced hyperalgesia [36]. Similar to opioid-induced hyperalgesia, patients may experience increased pain sensitivity and decreased pain tolerance with long-term exposure to CBMPs. However, further research and longer-term studies are warranted in studying this phenomenon. Alternatively, repeated CBMP exposure may diminish patients’ perceived benefits over time, in keeping with the subjective nature of PROMs. Furthermore, using the baseline observations carried forward method for missing data could also contribute to this effect.

The naturalistic design of this study allowed patients to continue their existing medications alongside CBMP treatment. Similarly, RCTs by Skrabek et al*.* and Chaves et al*.* both permitted patients to continue their ongoing pain therapies [17, 18], emphasising the role of CBMPs as an adjunct to multimodal treatment. A cross-sectional study found that 66% of fibromyalgia patients use alternative and complementary therapies [37], reporting greater pain relief than those using pharmacologic treatments alone. Preclinical studies suggest that CBMPs may work synergistically with pain-relief medications such as opioids, potentially reducing required dosages [38]. Supporting this, an observational study noted that among fibromyalgia patients taking CBMPs over 6 months, 20.3% reduced their benzodiazepine dosage, and 22.2% stopped or reduced their opioid dosage [39].

There are limited comparisons of the effectiveness of CBMPs against other medications prescribed for fibromyalgia, such as tricyclic antidepressants and serotonin-norepinephrine reuptake inhibitors. However, a 2-week double-blind placebo-controlled trial aimed to compare nabilone to amitriptyline in fibromyalgia patients with chronic insomnia [19]. In this study, there was no difference between groups on pain or quality of life. Moreover, nabilone was shown to have greater effects on self-reported sleep quality [19]. The limited nature of this study means that further assessment with well-powered RCTs, with sufficient follow-up, is needed to assess the relative efficacy of CBMPs against current therapeutics.

This present study identified short- to medium-term improvements in sleep, anxiety, and general quality of life, with regard to the SQS, GAD-7, and EQ-5D-5L scales respectively. This is in accordance with preclinical data that CBMPs have hypnotic and anxiolytic benefits [10], as well as analgesic effects [9]. An RCT by Ware et al*.* found nabilone was superior to amitriptyline for improving sleep in fibromyalgia patients in a short-term period [19], though both had a similar effect on pain and mood. Sleep and chronic pain have a bidirectional relationship, wherein pain can lead to poor sleep, and poor sleep can lead to increased pain [40]. Addressing each simultaneously may therefore have supplementary benefits on pain severity and its impact on quality of life.

### Adverse events

The high incidence of AEs relative to other studies from the UKMCR may reflect the central sensitisation mechanism in fibromyalgia, where increased dorsal horn neuron excitability could enhance responsiveness to new stimuli, such as CBMPs, and even formerly innocuous stimuli [21, 34, 41]. Fibromyalgia patients report more AEs than those with inflammatory arthritis and non-painful controls [42]. A meta-analysis of 2026 placebo-controlled patients found 67.2% reported one or more AEs, and 9.5% discontinued placebo treatment due to intolerance, suggesting a strong nocebo effect [43]. Furthermore, research suggests that females report more AEs than males [44], which is important to consider given that 68.61% of patients in this study were female.

### Limitations

As a case series, this study cannot determine causality, and whether the observed improvements were due to CBMPs alone and not confounding factors, such as lifestyle changes or concurrent treatments. This study was uncontrolled, leaving uncertainty as to whether the improvements were attributed to placebo effects. Although there are placebo-controlled RCTs supporting CBMPs, they are challenging due to the distinct psychoactive effect of THC, meaning patients could potentially distinguish between placebo and CBMPs. There may be sampling bias as most patients (68.61%) had previously used cannabis, and perhaps already identified themselves to be treatment-responsive, exaggerating improvements. This could explain how patients were more likely to experience an improvement in fibromyalgia-specific scales if they were a current user of cannabis. Furthermore, most patients were female (68.61,%); however, this is in line with typical patient demographics for fibromyalgia [2].

The high rates of co-morbidities in fibromyalgia make it difficult to ascertain if clinical improvements were due to the effects of CBMPs on comorbid conditions, such as anxiety or depression, or fibromyalgia itself [4]. Data were also not collected on whether patients were compliant with their medication. It was not determined if the findings were clinically significant due to a lack of literature for an appropriate minimum clinically important difference for Fibromyalgia SS and WPI scales. Patients were only eligible to receive CBMP treatment if they were resistant to conventional licensed medication [25], limiting the generalisability of the results to a general fibromyalgia population.

PROMs are subjective and susceptible to recall bias. Questionnaires were completed online, potentially causing a selection bias by limiting the inclusion of those less proficient with technology. There may also be selection bias with regard to socioeconomic status, as data originated from a private clinic. However, the majority of patients (53.92%) were unemployed. Positive media coverage of CBMPs may introduce expectancy bias [45], overestimating improvements. This bias may also lead to under-reporting of AEs. AEs were not confirmed to be treatment related. The UKMCR is susceptible to errors in coding, such as during data transfer from patient care summaries.

## Conclusion

This study found treatment with CBMPs in fibromyalgia was associated with short to medium-term improvements in pain, anxiety, sleep, and general quality of life. There was a high incidence of AEs, perhaps due to its central sensitisation mechanism, associated with an increased susceptibility to AEs. However, these findings must be interpreted within the limitations of the study design. More randomised controlled trials are warranted, but this large analysis provides real-world data to inform their conduct.

## Supplementary information

Below is the link to the electronic supplementary material.

Supplementary file 1 (DOCX 65.8 KB)

## Data Availability

Data that support the findings of this study are available from the UK Medical Cannabis Registry. Restrictions apply to the availability of these data. Data specifications and applications are available from the corresponding author. All authors contributed to and approved the final article. All work was conducted at Curaleaf Clinic, London, UK.
