# Supplementary material for: UK Medical Cannabis Registry: a case series analysing clinical outcomes of medicinal cannabis therapy for fibromyalgia
Source: Clin Rheumatol. 2025 Dec 4;45(3):1927–38. doi: 10.1007/s10067-025-07846-6 (PMC12923461; doi:10.1007/s10067-025-07846-6)
Supplement: Supplementary file 1 — (DOCX 65.8 KB) [file 10067_2025_7846_MOESM1_ESM.docx]

**Appendix A**: Prevalence of comorbidities in participants at baseline. *Abbreviations: AIDS – acquired immunodeficiency syndrome*

| **Baseline Patient Comorbidities** | **n (%)** |
| --- | --- |
| **Myocardial Infarction** | 8 (1.61) |
| **Congestive Heart Failure** | 2 (0.40) |
| **Leukaemia** | 1 (0.20) |
| **Peripheral Vascular Disease** | 0 (0.00) |
| **Endocrine Thyroid Dysfunction** | 47 (9.46) |
| **Depression or Anxiety** | 263 (52.92) |
| **Venous Thromboembolism** | 9 (1.81) |
| **Epilepsy** | 9 (1.81) |
| **Hypertension** | 52 (10.46) |
| **Arthritis** | 127 (25.55) |
| **AIDS** | 0 (0.00) |
| **Lymphoma** | 0 (0.00) |
| **Cerebrovascular Accident or Transient Ischaemic Attack** | 5 (1.01) |
| **Connective Tissue Disease** | 86 (17.30) |
| **Peptic Ulcer Disease** | 13 (2.62) |
| **Moderate to Severe Chronic Kidney Disease** | 11 (2.21) |
| **Hemiplegia** | 1 (0.20) |
| **Dementia** | 0 (0.00) |
| **Chronic Obstructive Pulmonary Disease** | 13 (2.62) |
| **Diabetes** |  |
| **-**       End organ damage | 2 (0.40) |
| **-**       None or diet-controlled | 472 (94.97) |
| **-**       Uncomplicated | 23 (4.63) |
| **Liver disease (mild)** | 17 (3.42) |
| **Solid tumor** |  |
| Localised | 12 (2.41) |
| Metastatic | 1 (0.20) |

**Appendix B**: Prevalence of patients in different geographical areas of the United Kingdom and Channel Islands.

| **Geographical Location** | **n (%)** |
| --- | --- |
| Channel Islands | 11 (2.21) |
| East Midlands | 27 (5.43) |
| East of England | 41 (8.25) |
| Isle of Man | 12 (2.41) |
| London | 63 (12.68) |
| North East | 20 (4.02) |
| North West | 47 (9.46) |
| Northern Ireland | 14 (2.82) |
| Scotland | 101 (20.32) |
| South East | 54 (10.87) |
| South West | 32 (6.44) |
| South Yorkshire | 8 (1.61) |
| Wales | 19 (3.82) |
| West Midlands | 30 (6.04) |
| Yorkshire and the Humber | 18 (3.82) |

**Appendix C**: An overview of adverse events observed during the study. *Abbreviations: %: percentage; S.D.: standard deviation*

|  | **Frequency by severity** | | | | **Overall frequency (%)** |
| --- | --- | --- | --- | --- | --- |
| **Adverse Event** | **Mild** | **Moderate** | **Severe** | **Life-threatening/disabling** |  |
| Abdominal pain | 34 | 26 | 8 | 0 | 68 (13.68) |
| Agitation | 0 | 0 | 1 | 0 | 1 (0.20) |
| Amnesia | 22 | 17 | 2 | 0 | 41 (8.25) |
| Anorexia | 19 | 21 | 2 | 0 | 42 (8.45) |
| Anxiety | 2 | 4 | 1 | 1 | 8 (1.61) |
| Appetite Increase | 3 | 0 | 0 | 0 | 3 (0.60) |
| Ataxia | 24 | 20 | 3 | 0 | 47 (9.46) |
| Blurred Vision | 24 | 16 | 3 | 0 | 43 (8.65) |
| Body Odour | 1 | 0 | 0 | 0 | 1 (0.20) |
| Bone Pain | 0 | 3 | 0 | 0 | 3 (0.60) |
| Chills | 0 | 0 | 1 | 0 | 1 (0.20) |
| Cognitive disturbance | 35 | 51 | 7 | 0 | 93 (18.71) |
| Concentration impairment | 55 | 62 | 13 | 0 | 130 (26.16) |
| Confusion | 32 | 23 | 5 | 0 | 60 (12.07) |
| Constipation | 50 | 29 | 9 | 0 | 88 (17.71) |
| Cough | 0 | 2 | 0 | 0 | 2 (0.40) |
| Delirium | 17 | 11 | 4 | 0 | 32 (6.44) |
| Depression | 0 | 2 | 2 | 0 | 4 (0.80) |
| Diarrhoea | 2 | 10 | 1 | 0 | 13 (2.62) |
| Dissociation | 0 | 1 | 1 | 0 | 2 (0.40) |
| Dizziness | 33 | 42 | 16 | 0 | 91 (18.31) |
| Dry mouth | 118 | 19 | 0 | 0 | 137 (27.57) |
| Dysaesthesia | 0 | 5 | 0 | 0 | 5 (1.01) |
| Dysgeusia | 0 | 13 | 8 | 0 | 21 (4.23) |
| Dyspepsia | 38 | 24 | 6 | 0 | 68 (13.68) |
| Dyspnoea | 0 | 1 | 0 | 0 | 1 (0.20) |
| Erectile dysfunction | 0 | 1 | 0 | 0 | 1 (0.20) |
| Euphoria | 0 | 1 | 0 | 0 | 1 (0.20) |
| Fall | 11 | 3 | 1 | 0 | 15 (3.02) |
| Fatigue | 14 | 75 | 64 | 0 | 153 (30.78) |
| Fever | 12 | 2 | 1 | 0 | 15 (3.02) |
| Flatulence | 0 | 1 | 0 | 0 | 1 (0.20) |
| Generalized muscle weakness | 14 | 33 | 28 | 0 | 75 (15.09) |
| Headache | 32 | 65 | 30 | 0 | 127 (25.55) |
| Hiccoughs | 0 | 1 | 0 | 0 | 1 (0.20) |
| Hypotension | 0 | 2 | 0 | 0 | 2 (0.40) |
| Insomnia | 18 | 39 | 38 | 0 | 95 (19.11) |
| Irritability | 0 | 1 | 0 | 0 | 1 (0.20) |
| Joint dislocation | 0 | 0 | 1 | 0 | 1 (0.20) |
| Lethargy | 28 | 101 | 0 | 0 | 129 (25.96) |
| Lung infection | 0 | 7 | 0 | 1 | 8 (1.61) |
| Memory impairment | 0 | 2 | 0 | 0 | 2 (0.40) |
| Migraine | 0 | 0 | 1 | 0 | 1 (0.20) |
| Myalgia | 1 | 1 | 0 | 0 | 2 (0.40) |
| Nausea | 58 | 28 | 1 | 0 | 87 (17.51) |
| Oedema Limbs | 2 | 0 | 0 | 0 | 2 (0.40) |
| Oral Pain | 1 | 1 | 0 | 0 | 2 (0.40) |
| Pain | 0 | 3 | 1 | 0 | 4 (0.80) |
| Paranoia | 1 | 3 | 0 | 0 | 4 (0.80) |
| Parasomnia | 0 | 1 | 1 | 0 | 2 (0.40) |
| Pharyngitis | 0 | 37 | 1 | 0 | 38 (7.65) |
| Pruritus | 0 | 1 | 0 | 0 | 1 (0.20) |
| Rash (non-specific) | 5 | 8 | 0 | 0 | 13 (2.62) |
| Seizure | 0 | 1 | 1 | 0 | 2 (0.40) |
| Somnolence | 0 | 95 | 24 | 0 | 119 (23.94) |
| Spasticity | 8 | 8 | 4 | 0 | 20 (4.02) |
| Tinnitus | 1 | 1 | 1 | 0 | 3 (0.60) |
| Tremor | 25 | 13 | 1 | 0 | 39 (7.85) |
| Urinary Retention | 1 | 0 | 0 | 0 | 1 (0.20) |
| Urinary Tract Infection | 0 | 9 | 1 | 0 | 10 (2.01) |
| Vertigo | 36 | 26 | 12 | 0 | 74 (14.89) |
| Vomiting | 10 | 5 | 1 | 0 | 16 (3.22) |
| Weight loss | 24 | 4 | 0 | 0 | 28 (5.63) |
| **Total** | 811 | 981 | 306 | 2 | 2100 |

**Appendix D**: Univariate logistic regression of an improvement in the Fibromyalgia Symptom Severity scale with multiple different variables. *Abbreviations: n: number of participants; CBMP: cannabis-based medicinal product; THC: tetrahydrocannabinol; CBD: cannabidiol; GAD-7: Generalised anxiety disorder scale; SQS: Sleep quality scale*

|  | **n** | **Odds ratio (95% Confidence Interval)** | ***p*-value** |
| --- | --- | --- | --- |
| **Gender** |  |  |  |
| Female | 309 | Ref | Ref |
| Male | 135 | 1.36 (0.88 – 2.11) | 0.167 |
| **Age (years)** |  |  |  |
| 18-30 | 49 | Ref | Ref |
| 31-40 | 138 | 1.41 (0.66 - 3.03) | 0.379 |
| 41-50 | 116 | 2.27 (1.05 – 4.89) | 0.036 |
| 51-60 | 94 | 1.06 (0.46 - 2.41) | 0.898 |
| 61-70 | 35 | 0.86 (0.30 - 2.51) | 0.788 |
| 71 + | 14 | 0.94 (0.22 - 3.99) | 0.935 |
| **Body Mass Index (kg/m2)** |  |  |  |
| <18.5 | 17 | 0.42 (0.12 - 1.56) | 0.195 |
| 18-5-24.9 | 125 | Ref | Ref |
| 25.0-29.9 | 112 | 0.76 (0.43 - 1.32) | 0.325 |
| 30-34.9 | 91 | 0.92 (0.52 - 1.64) | 0.789 |
| 35-39.9 | 43 | 1.17 (0.57 - 2.41) | 0.668 |
| ≥ 40.0 | 41 | 0.48 (0.20 - 1.13) | 0.092 |
| **Cannabis Status** |  |  |  |
| Never used | 142 | Ref | Ref |
| Current user | 236 | 2.91 (1.73-4.90) | <0.001 |
| Ex-user | 68 | 2.31 (1.17-4.57) | 0.016 |
| **CBMP Method of Administration** |  |  |  |
| Oils | 132 | Ref | Ref |
| Flower | 85 | 3.19 (1.68 – 6.05) | <0.001 |
| Both | 226 | 2.57 (1.50 - 4.43) | <0.001 |
| **Total THC Dosage (mg/day)** |  |  |  |
| ≤ median dose (112.50) | 232 | Ref | Ref |
| > median dose (112.50) | 214 | 2.45 (1.60-3.73) | <0.001 |
| **Total CBD Dosage (mg/day)** |  |  |  |
| ≤ median dose (25.00) | 256 | Ref | Ref |
| > median dose (25.00) | 190 | 1.95 (1.29-2.95) | 0.002 |
| **Baseline GAD-7 Score** |  |  |  |
| 0-4 | 118 | Ref | Ref |
| 5-9 | 120 | 1.25 (0.71-2.18) | 0.442 |
| 10-14 | 97 | 1.26 (0.70-2.27) | 0.438 |
| ≥15 | 111 | 0.91 (0.50-1.64) | 0.745 |
| **Baseline SQS Score** |  |  |  |
| 0-3 | 259 | 1.10 (0.72-1.67) | 0.659 |
| 4-10 | 187 | Ref | Ref |

**Appendix E**: Multivariate logistic regression of an improvement in the Fibromyalgia Symptom Severity scale with multiple different variables. *Abbreviations: n: number of participants; CBMP: cannabis-based medicinal product; THC: tetrahydrocannabinol; CBD: cannabidiol; GAD-7: Generalised anxiety disorder scale; SQS: Sleep quality scale*

|  | **n** | **Odds ratio (95% Confidence Interval)** | ***p*-value** |
| --- | --- | --- | --- |
| **Gender** |  |  |  |
| Female | 298 | Ref | Ref |
| Male | 127 | 1.02 (0.62 - 1.69) | 0.941 |
| **Age (years)** |  |  |  |
| 18-30 | 45 | Ref | Ref |
| 31-40 | 131 | 1.32 (0.58 - 3.00) | 0.510 |
| 41-50 | 113 | 2.29 (0.99 - 5.30) | 0.052 |
| 51-60 | 91 | 1.29 (0.53 - 3.15) | 0.572 |
| 61-70 | 33 | 1.89 (0.59 - 6.10) | 0.285 |
| 71 + | 12 | 2.11 (0.43 - 10.40) | 0.358 |
| **Body Mass Index (kg/m2)** |  |  |  |
| <18.5 | 17 | 0.35 (0.09 - 1.34) | 0.125 |
| 18-5-24.9 | 124 | Ref | Ref |
| 25.0-29.9 | 111 | 0.70 (0.38 - 1.27) | 0.238 |
| 30-34.9 | 89 | 1.01 (0.53 - 1.93) | 0.967 |
| 35-39.9 | 43 | 1.21 (0.54 - 2.69) | 0.646 |
| ≥ 40.0 | 41 | 0.41 (0.16 - 1.02) | 0.056 |
| **Cannabis Status** |  |  |  |
| Never used | 138 | Ref | Ref |
| Current user | 222 | 2.32 (1.22 - 4.43) | 0.011 |
| Ex-user | 65 | 2.22 (1.04 - 4.75) | 0.040 |
| **CBMP Method of Administration** |  |  |  |
| Oils | 123 | Ref | Ref |
| Flower | 83 | 1.39 (0.60 - 3.25) | 0.442 |
| Both | 219 | 1.14 (0.54 - 2.41) | 0.728 |
| **Total THC Dosage (mg/day)** |  |  |  |
| ≤ median dose (112.50) | 216 | Ref | Ref |
| > median dose (112.50) | 209 | 1.52 (0.85 - 2.73) | 0.162 |
| **Total CBD Dosage (mg/day)** |  |  |  |
| ≤ median dose (25.00) | 241 | Ref | Ref |
| > median dose (25.00) | 184 | 1.64 (1.04 - 2.61) | 0.035 |
| **Baseline GAD-7 Score** |  |  |  |
| 0-4 | 116 | Ref | Ref |
| 5-9 | 115 | 1.28 (0.68 - 2.39) | 0.442 |
| 10-14 | 89 | 1.19 (0.61 - 2.32) | 0.602 |
| ≥15 | 105 | 0.90 (0.45 - 1.77) | 0.752 |
| **Baseline SQS Score** |  |  |  |
| 0-3 | 245 | 0.96 (0.59 - 1.55) | 0.866 |
| 4-10 | 180 | Ref | Ref |

**Appendix F**: Univariate logistic regression of an improvement in the Fibromyalgia Widespread Pain Index scale with multiple different variables. *Abbreviations: n: number of participants; CBMP: cannabis-based medicinal product; THC: tetrahydrocannabinol; CBD: cannabidiol; GAD-7: Generalised anxiety disorder scale; SQS: Sleep quality scale*

|  | **n** | **Odds ratio (95% Confidence Interval)** | ***p*-value** |
| --- | --- | --- | --- |
| **Gender** |  |  |  |
| Female | 309 | Ref | Ref |
| Male | 135 | 1.36 (0.88 – 2.11) | 0.167 |
| **Age (years)** |  |  |  |
| 18-30 | 49 | Ref | Ref |
| 31-40 | 138 | 1.57 (0.69 - 3.55) | 0.280 |
| 41-50 | 116 | 3.37 (1.50 - 7.58) | 0.003 |
| 51-60 | 94 | 1.52 (0.65 - 3.60) | 0.336 |
| 61-70 | 35 | 1.11 (0.37 - 3.34) | 0.851 |
| 71 + | 14 | 0.74 (0.14 - 3.91) | 0.723 |
| **Body Mass Index (kg/m2)** |  |  |  |
| <18.5 | 17 | 0.70 (0.22 - 2.30) | 0.562 |
| 18-5-24.9 | 125 | Ref | Ref |
| 25.0-29.9 | 112 | 0.84 (0.48 - 1.47) | 0.539 |
| 30-34.9 | 91 | 1.02 (0.57 - 1.83) | 0.954 |
| 35-39.9 | 43 | 1.36 (0.66 - 2.81) | 0.410 |
| ≥ 40.0 | 41 | 0.95 (0.44 - 2.05) | 0.891 |
| **Cannabis Status** |  |  |  |
| Never used | 142 | Ref | Ref |
| Current user | 236 | 2.81 (1.67 - 4.72) | <0.001 |
| Ex-user | 68 | 2.47 (1.26 - 4.87) | 0.009 |
| **CBMP Method of Administration** |  |  |  |
| Oils | 132 | Ref | Ref |
| Flower | 85 | 2.20 (1.13 - 4.31) | 0.021 |
| Both | 226 | 3.25 (1.88 – 5.62) | <0.001 |
| **Total THC Dosage (mg/day)** |  |  |  |
| ≤ median dose (112.50) | 232 | Ref | Ref |
| > median dose (112.50) | 214 | 2.76 (1.80 – 4.23) | <0.001 |
| **Total CBD Dosage (mg/day)** |  |  |  |
| ≤ median dose (25.00) | 256 | Ref | Ref |
| > median dose (25.00) | 190 | 2.39 (1.57 - 3.63) | <0.001 |
| **Baseline GAD-7 Score** |  |  |  |
| 0-4 | 118 | Ref | Ref |
| 5-9 | 120 | 1.02 (0.59 - 1.76) | 0.957 |
| 10-14 | 97 | 1.12 (0.63 - 2.00) | 0.697 |
| ≥15 | 111 | 0.60 (0.33 - 1.09) | 0.092 |
| **Baseline SQS Score** |  |  |  |
| 0-3 | 259 | 0.90 (0.60 - 1.36) | 0.621 |
| 4-10 | 187 | Ref | Ref |

**Appendix G**: Multivariate logistic regression of an improvement in the Fibromyalgia Widespread Pain Index scale with multiple different variables. *Abbreviations: n: number of participants; CBMP: cannabis-based medicinal product; THC: tetrahydrocannabinol; CBD: cannabidiol; GAD-7: Generalised anxiety disorder scale; SQS: Sleep quality scale*

|  | **n** | **Odds ratio (95% Confidence Interval)** | ***p*-value** |
| --- | --- | --- | --- |
| **Gender** |  |  |  |
| Female | 298 | Ref | Ref |
| Male | 127 | 0.90 (0.54 - 1.50) | 0.674 |
| **Age (years)** |  |  |  |
| 18-30 | 45 | Ref | Ref |
| 31-40 | 131 | 1.47 (0.61 - 3.58) | 0.393 |
| 41-50 | 113 | 3.39 (1.38 - 8.31) | 0.008 |
| 51-60 | 91 | 2.02 (0.79 - 5.16) | 0.144 |
| 61-70 | 33 | 2.46 (0.73 - 8.36) | 0.148 |
| 71 + | 12 | 1.42 (0.23 - 8.89) | 0.710 |
| **Body Mass Index (kg/m2)** |  |  |  |
| <18.5 | 17 | 0.54 (0.15 - 1.88) | 0.332 |
| 18-5-24.9 | 124 | Ref | Ref |
| 25.0-29.9 | 111 | 0.81 (0.44 - 1.52) | 0.518 |
| 30-34.9 | 89 | 1.15 (0.59 - 2.25) | 0.675 |
| 35-39.9 | 43 | 1.84 (0.81 - 4.22) | 0.148 |
| ≥ 40.0 | 41 | 0.87 (0.37 - 2.07) | 0.758 |
| **Cannabis Status** |  |  |  |
| Never used | 138 | Ref | Ref |
| Current user | 222 | 2.32 (1.21 - 4.45) | 0.011 |
| Ex-user | 65 | 2.63 (1.22 - 5.68) | 0.014 |
| **CBMP Method of Administration** |  |  |  |
| Oils | 123 | Ref | Ref |
| Flower | 83 | 0.81 (0.33 - 1.97) | 0.639 |
| Both | 219 | 1.44 (0.67 - 3.06) | 0.347 |
| **Total THC Dosage (mg/day)** |  |  |  |
| ≤ median dose (112.50) | 216 | Ref | Ref |
| > median dose (112.50) | 209 | 1.70 (0.93 - 3.09) | 0.084 |
| **Total CBD Dosage (mg/day)** |  |  |  |
| ≤ median dose (25.00) | 241 | Ref | Ref |
| > median dose (25.00) | 184 | 1.90 (1.19 - 3.03) | 0.007 |
| **Baseline GAD-7 Score** |  |  |  |
| 0-4 | 116 | Ref | Ref |
| 5-9 | 115 | 1.03 (0.55 - 1.93) | 0.932 |
| 10-14 | 89 | 1.10 (0.56 - 2.13) | 0.787 |
| ≥15 | 105 | 0.63 (0.31 - 1.29) | 0.207 |
| **Baseline SQS Score** |  |  |  |
| 0-3 | 245 | 0.79 (0.49 - 1.29) | 0.350 |
| 4-10 | 180 | Ref | Ref |

**Appendix H**: Univariate logistic regression of an improvement in the Fibromyalgia scale overall with multiple different variables. *Abbreviations: n: number of participants; CBMP: cannabis-based medicinal product; THC: tetrahydrocannabinol; CBD: cannabidiol; GAD-7: Generalised anxiety disorder scale; SQS: Sleep quality scale*

|  | **n** | **Odds ratio (95% Confidence Interval)** | ***p*-value** |
| --- | --- | --- | --- |
| **Gender** |  |  |  |
| Female | 309 | Ref | Ref |
| Male | 135 | 1.26 (0.82 - 1.94) | 0.299 |
| **Age (years)** |  |  |  |
| 18-30 | 49 | Ref | Ref |
| 31-40 | 138 | 1.27 (0.59 - 2.73) | 0. 549 |
| 41-50 | 116 | 2.91 (1.35 - 6.24) | 0.006 |
| 51-60 | 94 | 1.32 (0.59 - 2.97) | 0.500 |
| 61-70 | 35 | 0.86 (0.30 - 2.51) | 0.788 |
| 71 + | 14 | 0.94 (0.22 - 3.99) | 0.935 |
| **Body Mass Index (kg/m2)** |  |  |  |
| <18.5 | 17 | 0.79 (0.26 - 2.40) | 0.684 |
| 18-5-24.9 | 125 | Ref | Ref |
| 25.0-29.9 | 112 | 0.80 (0.46 - 1.38) | 0.417 |
| 30-34.9 | 91 | 0.89 (0.50 - 1.59) | 0.697 |
| 35-39.9 | 43 | 1.25 (0.61 - 2.55) | 0.545 |
| ≥ 40.0 | 41 | 0.62 (0.28 - 1.37) | 0.235 |
| **Cannabis Status** |  |  |  |
| Never used | 142 | Ref | Ref |
| Current user | 236 | 2.73 (1.65 - 4.53) | <0.001 |
| Ex-user | 68 | 2.72 (1.41 - 5.24) | 0.003 |
| **CBMP Method of Administration** |  |  |  |
| Oils | 132 | Ref | Ref |
| Flower | 85 | 2.92 (1.50 – 5.67) | 0.002 |
| Both | 226 | 3.86 (2.22 – 6.73) | <0.001 |
| **Total THC Dosage (mg/day)** |  |  |  |
| ≤ median dose (112.50) | 232 | Ref | Ref |
| > median dose (112.50) | 214 | 2.86 (1.88 - 4.35) | <0.001 |
| **Total CBD Dosage (mg/day)** |  |  |  |
| ≤ median dose (25.00) | 256 | Ref | Ref |
| > median dose (25.00) | 190 | 2.90 (1.92 – 4.39) | <0.001 |
| **Baseline GAD-7 Score** |  |  |  |
| 0-4 | 118 | Ref | Ref |
| 5-9 | 120 | 1.18 (0.69 - 2.04) | 0.547 |
| 10-14 | 97 | 1.23 (0.69 - 2.18) | 0.480 |
| ≥15 | 111 | 0.70 (0.39 - 1.26) | 0.229 |
| **Baseline SQS Score** |  |  |  |
| 0-3 | 259 | 1.02 (0.68 - 1.53) | 0.927 |
| 4-10 | 187 | Ref | Ref |

**Appendix I**: Multivariate logistic regression of an improvement in the Fibromyalgia scale overall with multiple different variables. *Abbreviations: n: number of participants; CBMP: cannabis-based medicinal product; THC: tetrahydrocannabinol; CBD: cannabidiol; GAD-7: Generalised anxiety disorder scale; SQS: Sleep quality scale*

|  | **n** | **Odds ratio (95% Confidence Interval)** | ***p*-value** |
| --- | --- | --- | --- |
| **Gender** |  |  |  |
| Female | 298 | Ref | Ref |
| Male | 127 | 1.02 (0.61 - 1.71) | 0.931 |
| **Age (years)** |  |  |  |
| 18-30 | 45 | Ref | Ref |
| 31-40 | 131 | 1.04 (0.45 - 2.42) | 0.928 |
| 41-50 | 113 | 2.95 (1.26 - 6.93) | 0.013 |
| 51-60 | 91 | 1.77 (0.72 - 4.33) | 0.213 |
| 61-70 | 33 | 2.04 (0.61 - 6.81) | 0.245 |
| 71 + | 12 | 2.12 (0.41 - 11.08) | 0.371 |
| **Body Mass Index (kg/m2)** |  |  |  |
| <18.5 | 17 | 0.65 (0.20 - 2.11) | 0.474 |
| 18-5-24.9 | 124 | Ref | Ref |
| 25.0-29.9 | 111 | 0.81 (0.44 - 1.49) | 0.503 |
| 30-34.9 | 89 | 1.05 (0.54 - 2.04) | 0.887 |
| 35-39.9 | 43 | 1.46 (0.65 - 3.32) | 0.361 |
| ≥ 40.0 | 41 | 0.52 (0.21 - 1.29) | 0.158 |
| **Cannabis Status** |  |  |  |
| Never used | 138 | Ref | Ref |
| Current user | 222 | 1.97 (1.04 - 3.73) | 0.038 |
| Ex-user | 65 | 2.91 (1.35 - 6.24) | 0.006 |
| **CBMP Method of Administration** |  |  |  |
| Oils | 123 | Ref | Ref |
| Flower | 83 | 1.44 (0.60 - 3.45) | 0.414 |
| Both | 219 | 2.02 (0.95 - 4.29) | 0.067 |
| **Total THC Dosage (mg/day)** |  |  |  |
| ≤ median dose (112.50) | 216 | Ref | Ref |
| > median dose (112.50) | 209 | 1.51 (0.85 - 2.71) | 0.163 |
| **Total CBD Dosage (mg/day)** |  |  |  |
| ≤ median dose (25.00) | 241 | Ref | Ref |
| > median dose (25.00) | 184 | 2.42 (1.51 - 3.86) | <0.001 |
| **Baseline GAD-7 Score** |  |  |  |
| 0-4 | 116 | Ref | Ref |
| 5-9 | 115 | 1.29 (0.68 - 2.43) | 0.431 |
| 10-14 | 89 | 1.14 (0.58 - 2.22) | 0.706 |
| ≥15 | 105 | 0.74 (0.37 - 1.49) | 0.400 |
| **Baseline SQS Score** |  |  |  |
| 0-3 | 245 | 0.90 (0.55 - 1.46) | 0.659 |
| 4-10 | 180 | Ref | Ref |

**Appendix J**: Univariate logistic regression for the odds of reporting an adverse event with multiple different variables. *Abbreviations: n: number of participants; CBMP: cannabis-based medicinal product; THC: tetrahydrocannabinol; CBD: cannabidiol; GAD-7: Generalised anxiety disorder scale; SQS: Sleep quality scale*

|  | **n** | **Odds ratio (95% Confidence Interval)** | ***p*-value** |
| --- | --- | --- | --- |
| **Gender** |  |  |  |
| Female | 341 | Ref | Ref |
| Male | 154 | 0.70 (0.45-1.09) | 0.113 |
| **Age (years)** |  |  |  |
| 18-30 | 55 | Ref | Ref |
| 31-40 | 155 | 0.49 (0.25 - 0.95) | 0.034 |
| 41-50 | 128 | 0.45 (0.23 - 0.90) | 0.024 |
| 51-60 | 106 | 0.61 (0.31 - 1.22) | 0.161 |
| 61-70 | 39 | 1.70 (0.74 - 3.91) | 0.208 |
| 71 + | 14 | 0.44 (0.11 - 1.77) | 0.248 |
| **Body Mass Index (kg/m2)** |  |  |  |
| <18.5 | 20 | 0.46 (0.13 - 1.64) | 0.229 |
| 18-5-24.9 | 136 | Ref | Ref |
| 25.0-29.9 | 131 | 0.94 (0.55 - 1.61) | 0.823 |
| 30-34.9 | 98 | 1.14 (0.64 - 2.01) | 0.657 |
| 35-39.9 | 50 | 1.11 (0.54 - 2.25) | 0.783 |
| ≥ 40.0 | 42 | 0.92 (0.42 - 2.00) | 0.824 |
| **Cannabis Status** |  |  |  |
| Never used | 156 | Ref | Ref |
| Current user | 264 | 0.41 (0.26-0.63) | <0.001 |
| Ex-user | 77 | 0.58 (0.32-1.06) | 0.077 |
| **CBMP Method of Administration** |  |  |  |
| Oils | 147 | Ref | Ref |
| Flower | 97 | 0.61 (0.35-1.06) | 0.080 |
| Both | 249 | 0.42 (0.27-0.65) | <0.001 |
| **Total THC Dosage (mg/day)** |  |  |  |
| ≤ median dose (112.50) | 252 | Ref | Ref |
| > median dose (112.50) | 245 | 0.71 (0.48-1.05) | 0.087 |
| **Total CBD Dosage (mg/day)** |  |  |  |
| ≤ median dose (25.00) | 282 | Ref | Ref |
| > median dose (25.00) | 215 | 1.43 (0.96-2.12) | 0.077 |
| **Baseline GAD-7 Score** |  |  |  |
| 0-4 | 132 | Ref | Ref |
| 5-9 | 130 | 1.06 (0.61 - 1.85) | 0.831 |
| 10-14 | 106 | 1.48 (0.84 - 2.60) | 0.175 |
| ≥15 | 129 | 1.12 (0.64 - 1.94) | 0.695 |
| **Baseline SQS Score** |  |  |  |
| 0-3 | 285 | 0.59 (0.40-0.88) | 0.010 |
| 4-10 | 211 | Ref | Ref |

**Appendix K**: Multivariate logistic regression for the odds of reporting an adverse event with multiple different variables. *Abbreviations: n: number of participants; CBMP: cannabis-based medicinal product; THC: tetrahydrocannabinol; CBD: cannabidiol; GAD-7: Generalised anxiety disorder scale; SQS: Sleep quality scale*

|  | **n** | **Odds ratio (95% Confidence Interval)** | ***p*-value** |
| --- | --- | --- | --- |
| **Gender** |  |  |  |
| Female | 327 | Ref | Ref |
| Male | 145 | 1.23 (0.74-2.06) | 0.420 |
| **Age (years)** |  |  |  |
| 18-30 | 50 | Ref | Ref |
| 31-40 | 147 | 0.48 (0.23-1.01) | 0.053 |
| 41-50 | 124 | 0.40 (0.18-0.88) | 0.023 |
| 51-60 | 102 | 0.50 (0.22-1.11) | 0.087 |
| 61-70 | 37 | 1.36 (0.52-3.58) | 0.529 |
| 71 + | 12 | 0.09 (0.01-0.84) | 0.034 |
| **Body Mass Index (kg/m2)** |  |  |  |
| <18.5 | 20 | 0.41 (0.11-1.56) | 0.189 |
| 18-5-24.9 | 135 | Ref | Ref |
| 25.0-29.9 | 129 | 0.82 (0.45-1.47) | 0.497 |
| 30-34.9 | 96 | 0.93 (0.49-1.77) | 0.828 |
| 35-39.9 | 50 | 0.87 (0.39-1.94) | 0.730 |
| ≥ 40.0 | 42 | 0.80 (0.34-1.89) | 0.615 |
| **Cannabis Status** |  |  |  |
| Never used | 151 | Ref | Ref |
| Current user | 249 | 0.46 (0.26-0.83) | 0.010 |
| Ex-user | 72 | 0.69 (0.34-1.38) | 0.296 |
| **CBMP Method of Administration** |  |  |  |
| Oils | 136 | Ref | Ref |
| Flower | 95 | 0.74 (0.33-1.67) | 0.467 |
| Both | 241 | 0.44 (0.22-0.90) | 0.025 |
| **Total THC Dosage (mg/day)** |  |  |  |
| ≤ median dose (112.50) | 233 | Ref | Ref |
| > median dose (112.50) | 239 | 1.29 (0.68-2.44) | 0.436 |
| **Total CBD Dosage (mg/day)** |  |  |  |
| ≤ median dose (25.00) | 265 | Ref | Ref |
| > median dose (25.00) | 207 | 2.28 (1.42-3.66) | <0.001 |
| **Baseline GAD-7 Score** |  |  |  |
| 0-4 | 129 | Ref | Ref |
| 5-9 | 124 | 1.23 (0.65-2.32) | 0.519 |
| 10-14 | 97 | 2.24 (1.16-4.32) | 0.017 |
| ≥15 | 122 | 1.94 (1.01-3.70) | 0.045 |
| **Baseline SQS Score** |  |  |  |
| 0-3 | 270 | 0.47 (0.29-0.75) | 0.002 |
| 4-10 | 202 | Ref | Ref |
